# Dangers of Morphine

**Published:** 1897-04

**Authors:** 


### DANGERS OF MORPHINE.

The virtues of opium as an anodyne have been known to the profession since time immemorial. Morphine is the oldest known active principal of opium, but morphine, unfortunately, is not a proprietary preparation and there is no way of making it conform to a uniform standard of strength and purity. Opium contains certain dangerous narcotizing and convulsive principles and much of the morphine in the market is contaminated by their presence.

The freedom and lack of thought with which a majority of physicans administer large doses of morphine to patients already weakened by severe pain or shock is reprehensible. Undoubtedly, death in many such cases is due, not so much to the disease, as to the impure morphine employed.

Do not give opiates where the vital powers have been reduced to a low ebb, and where you must use them, choose the standard, ethpharmal preparations, whose makers are responsible for their purity.—*Medical Brief.*

